# Facile Synthesis of Flower-Like Copper-Cobalt Sulfide as Binder-Free Faradaic Electrodes for Supercapacitors with Improved Electrochemical Properties

**DOI:** 10.3390/nano7060140

**Published:** 2017-06-07

**Authors:** Tianlei Wang, Meitang Liu, Hongwen Ma

**Affiliations:** Beijing Key Laboratory of Materials Utilization of Nonmetallic Minerals and Solid Wastes, National Laboratory of Mineral Materials, School of Materials Science and Technology, China University of Geosciences, Beijing 100083, China; wangtl@cugb.edu.cn

**Keywords:** copper-cobalt sulfide, electrode material, hydrothermal method, binder-free

## Abstract

Supercapacitors have been one of the highest potential candidates for energy storage because of their significant advantages beyond rechargeable batteries in terms of large power density, short recharging time, and long cycle lifespan. In this work, Cu–Co sulfides with uniform flower-like structure have been successfully obtained via a traditional two-step hydrothermal method. The as-fabricated Cu–Co sulfide vulcanized from precursor (P–Cu–Co sulfide) is able to deliver superior specific capacitance of 592 F g^−1^ at 1 A g^−1^ and 518 F g^−1^ at 10 A g^−1^ which are surprisingly about 1.44 times and 2.39 times higher than those of Cu–Co oxide electrode, respectively. At the same time, excellent cycling stability of P–Cu–Co sulfide is indicated by 90.4% capacitance retention at high current density of 10 A g^−1^ after 3000 cycles. Because of the introduction of sulfur during the vulcanization process, these new developed sulfides can get more flexible structure and larger reaction surface area, and will own richer redox reaction sites between the interfaces of active material/electrolyte. The uniform flower-like P–Cu–Co sulfide electrode materials will have more potential alternatives for oxides electrode materials in the future.

## 1. Introduction

With the urgent targets to deal with the crisis of energy depletion, enthusiastic exploration of the environmental and efficient energy materials are engaged [1,2,3]. Supercapacitors, as a type of newly-emerging energy storage devices, have attracted tremendous attention for their ultrahigh power density and excellent electrochemical stability [4,5,6,7,8,9]. Therefore, lots of research has been conducted to improve the energy density under the precondition of maintaining their power density and long lifespan [10,11,12,13,14,15].

It is well-known that cobalt oxides are putative promising materials as electrodes with outstanding electrochemical property [16,17,18,19]. However, the single-metal oxides are sometimes limited to their broad application which are suffering from poor rate capability, poor charge-discharge reversibility, high cost for their raw materials, and high toxicity [20,21]. Sorts of attempts have been implemented to seek eco-friendly and less expensive metal element (such as Cu, Zn, Ni, Mg, and Fe) to replace the toxic and expensive Co aiming at the development of low cost and sustainable electrodes [21,22,23,24,25,26,27]. MnCo_2_O_4_, synthesized via facile hydrothermal method, has been designed as an energy storage device which displays excellent electrochemical properties [28]. As well, ternary metal oxides with nanowires [29], nanosheets [30,31], hollow spheres [32], core/shell [25,32,33,34,35], and flower-like [36,37] structures directly arraying on Ni foam exhibit significantly enhanced electrochemical performance.

Copper has been considered as a fascinating candidate instead of cobalt, due to its abundant natural resources, low cost, excellent chemical stability, and environmentally friendly capability. Due to its lots of advantages for electrochemical sensing, a sequence-specific DNA electrochemical biosensor was designed successfully based on CuS nanosheets [38]. Wang et al. reported core@shell CuCo_2_O_4_@MnO_2_ nanoarchitectures with a heightening specific capacitance, outstanding rate capability, and long-term lifespan in different bent states [25]. At the same time, novel and efficient flexible electrodes based on CuCo_2_O_4_ nanowires grown on Ni wire exhibit outstanding cycling stability and excellent flexible feature, which will make it possible to design wearable electronic devices [39].

In particular, notable transition metal sulfide electrode materials with high electrochemical activity have attracted amount of attention. Generally, due to the lower band gap, transition metal sulfides may exhibit much higher conductivity and lower electronegativity than corresponding transition metal oxides [40,41,42]. Ni@rGO-Co_3_S_4_ and Ni@rGO-Ni_3_S_2_ were synthesized as electrodes of advanced aqueous asymmetric supercapacitors, which are beneficial for improving the energy density accompanied by high cycle stability [43]. Yu et al. fabricated Ni_x_Co_3−x_S_4_ electrodes with hollow prisms microstructure via self-templating method, which exhibit high specific capacitance, desirable rate capability and good capacitance retention [26]. In our previous work, we successfully synthesized flower-like Mn–Co oxysulfide in which oxygen element in Mn–Co oxide is partially substituted by sulphur [37]. Recently, hierarchical CuCo_2_S_4_ hollow nanoneedles are synthesized via template-free hydrothermal method and designed as notable high-performance electrodes with superior electrochemical performances [44].

Based on the above considerations, we prepared the uniform flower-like Cu–Co sulfides arraying on Ni foam by traditional hydrothermal method for the first time. In particular, after making a comparison between Cu–Co oxides and Cu–Co sulfides which are respectively treated from Cu–Co precursor or Cu–Co oxide, we conclude the specific capacitance and rate capability of Cu–Co sulfides are superior to those of Cu–Co oxides. Besides, Cu–Co sulfide by vulcanizing from precursor has advantages over Cu–Co sulfide from oxide in those aspects. As a result, hopeful electrodes with fancy specific capacitance, wonderful rate capability, and interesting long-term cycling stability were synthesized, which is expected to be potential candidates for designing, constructing, and developing novel energy storage devices.

## 2. Results

**Figure 1:**
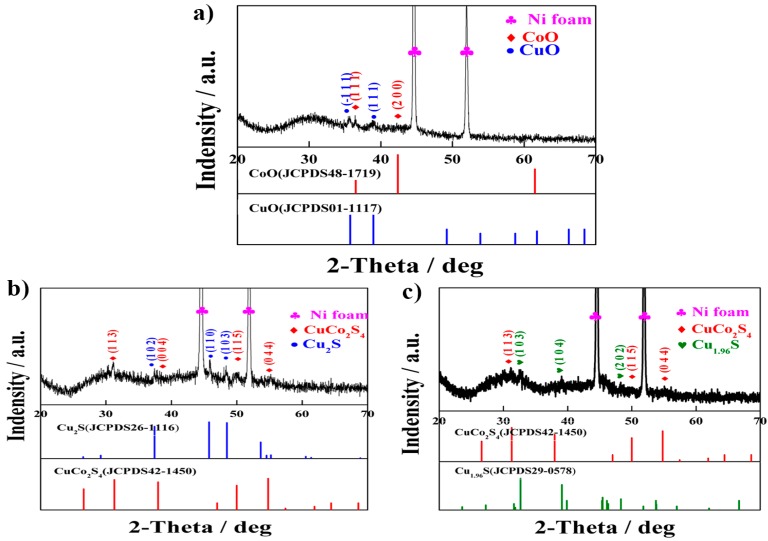
XRD patterns of sample on nickel foam, (**a**) Cu–Co oxide; (**b**) P–Cu–Co sulfide; (**c**) O–Cu–Co sulfide.

Regardless of the two highest peaks indicating Ni foam, all the diffraction peaks in Figure 1a can be well indexed as CoO (JCPDS 48-1719) and CuO (JCPDS 01-1117), which indicate that the Cu–Co oxides is polycrystalline. After Cu–Co precursor being vulcanized by Na_2_S·9H_2_O, four peaks corresponding to the (113), (004), (115), and (044) diffraction planes are in accordance with the cubic phase of CuCo_2_S_4_ (JCPDS 42-1450). The other diffraction peaks about (102), (110), and (103) can be well indexed to the standard X-Ray Diffraction (XRD) patterns of Cu_2_S (JCPDS 26-1116). It is manifested that the Cu–Co precursor is completely vulcanized and transformed into the polycrystalline CuCo_2_S_4_ and Cu_2_S (Figure 1b). As for O–Cu–Co sulfide, Cu_1.96_S (JCPDS 29-0578) whose diffraction peaks correspond to the (103), (104), and (202) diffraction planes are accompanied with the phase of CuCo_2_S_4_, indicating sulphur can entirely take the place of oxygen in the Cu–Co oxides during the vulcanizing process, and finally polycrystalline CuCo_2_S_4_ and Cu_1.96_S sulfides can be obtained (Figure 1c).

**Figure 2:**
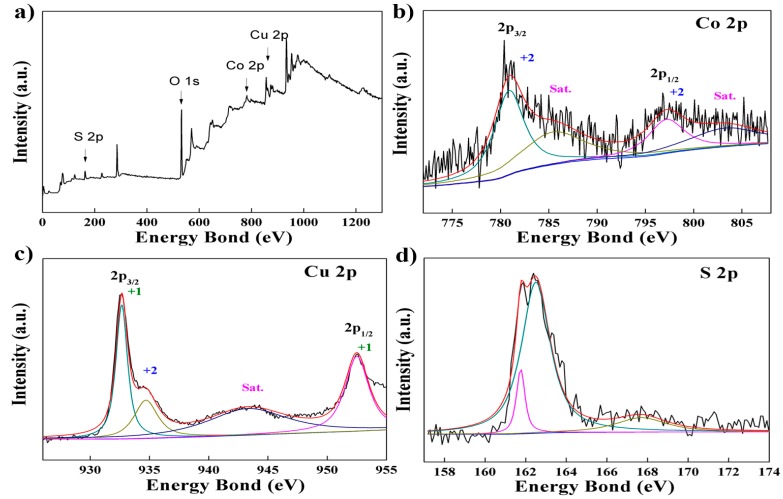
(**a**) The full-survey scan spectrum of P–Cu–Co sulfide; (**b**) high resolution spectra of Co 2p; (**c**) high resolution spectra of Cu 2p; (**d**) high resolution spectra of S 2p.

**Figure 3:**
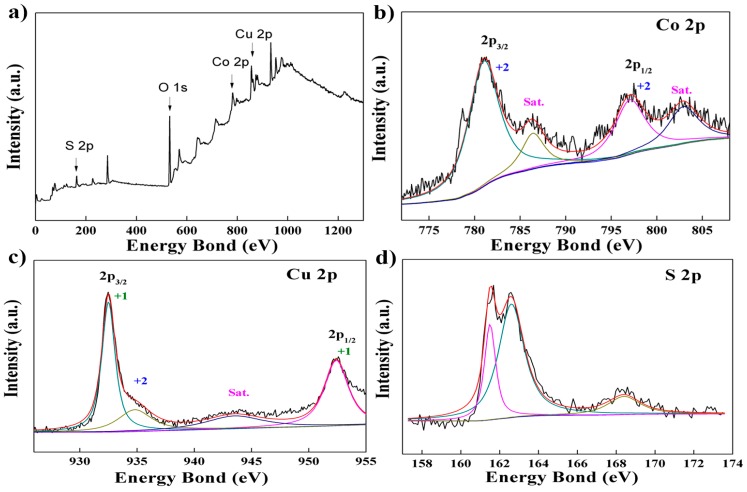
(**a**) The full-survey scan spectrum of O–Cu–Co sulfide; (**b**) high resolution spectra of Co 2p; (**c**) high resolution spectra of Cu 2p; (**d**) high resolution spectra of S 2p.

In Figure 2a and Figure 3a, the presence of Cu, Co, and S elements in the Cu–Co sulfides are indicated in the full-survey scan spectrum. Meanwhile, O 1s peaks can also be found because the surface of samples were oxidized in the air [45]. According to XPS spectrum for Co 2p in Figure 2b and Figure 3b, the binding energies at 781.0 and 797.2 eV of Co 2p_3/2_ and 2p_1/2_ peaks accompanied with two satellite peaks at around 786.4 and 802.9 eV are found, which demonstrate the existence of Co^2+^. Two peaks at binding energies of 932.5 and 952.4 eV (ΔE = 19.9 eV) are shown in Figure 2c and Figure 3c which corresponds to the Cu 2p_3/2_ and 2p_1/2_ matched with Cu^0^ or Cu^1+^. According to XRD analysis, the pure copper does not appear in P–Cu–Co sulfide and O–Cu–Co sulfide, so the copper element exists as Cu^1+^. At the same time, there is a weak peak at binding energies of 934.8 eV with a satellite peak at 943.3 eV, which indicate the Cu^2+^ [46,47]. As for the S 2p XPS spectrum (in Figure 2d and Figure 3d), the major peak at the binding energy of 162.5 eV coupled with satellite peak attribute to metal-sulfur bonding (Cu–S and Co–S bonding) [40]. Meanwhile, the binding energy centered at 168.0 eV matches with S–O bonding due to the oxidation effect on the samples in air. The XPS results, being consistent with the XRD results, indicate that the vulcanizing process is effective for Cu–Co compounds.

**Figure 4:**
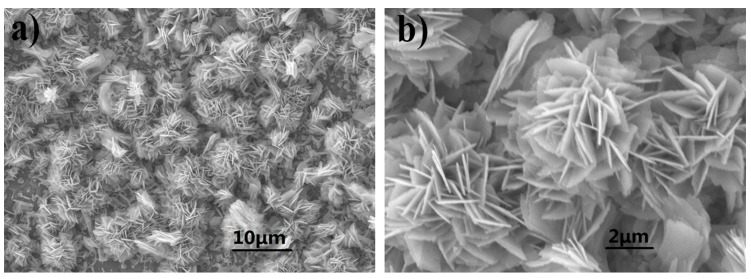

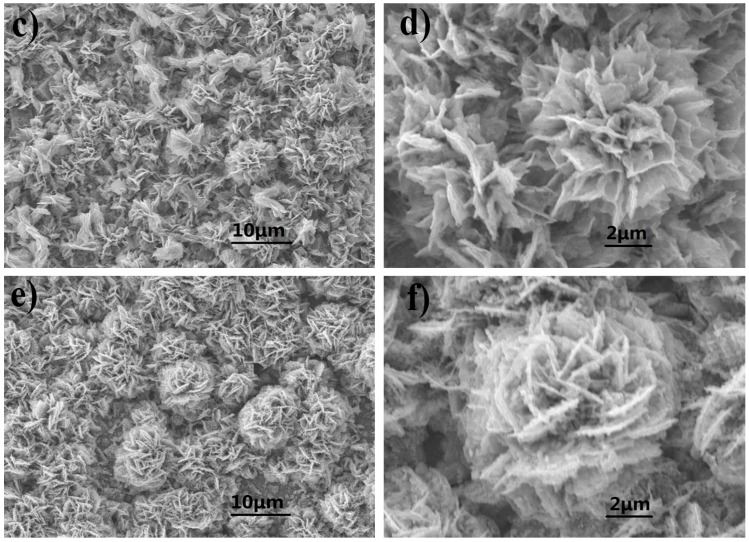
(**a**,**b**) SEM images of Cu–Co oxide; (**c**,**d**) SEM images of P–Cu–Co sulfide; (**e**,**f**) SEM images of O–Cu–Co sulfide.

Figure 4 exhibits the Scanning Electron Microscope (SEM) images of Cu–Co oxide (Figure 4a,b), P–Cu–Co sulfide (Figure 4c,d) and O–Cu–Co sulfide (Figure 4e,f) supported on nickel foam. Cu–Co oxides and sulfides nanoparticles evenly array on the Ni foam with flower-like structures. As seen from Figure 4b, the uniform flower-like structure of Cu–Co oxide is stacked by interconnecting nanosheets in different orientations, and the surfaces of nanosheets are regular and smooth. The average diameter of flower structure is about 6.5 μm and the nanosheets’ thickness is about 160 nm. Normally, the contact area with the perfect flower-like structure between active material and the electrolyte must be much larger than that of other structures. As a result, the uniform flower-like structure must be more suitable for Faradaic reaction. For P–Cu–Co sulfide, the edges of nanosheets are dimmed, and every nanosheet seems to be crosslinked to make the petal grown after sulfuration. The average diameter of flowers grow up to 10.5 μm. On the contrary, O–Cu–Co sulfide also displays the flower-like structure, but plenty of circular particles appear on the regular and smooth nanosheets. During the vulcanization of Cu–Co precursor without any calcining whose nanosheets have not yet fully formed, OH^−^ in precursor are substituted by S^2−^ leading to the flower growing larger and the nanosheets dimming. In contrast, Cu–Co oxide has stable structure after calcination, and the nanosheets do not change when S^2−^ replaces O^2−^ during the vulcanization process, only some spherical particles appear on it. Therefore, the flower-like morphology of Cu–Co sulfides provides vast channels and large surface areas for electrolyte ions which can make more efficient use of the electrode active materials.

**Figure 5:**
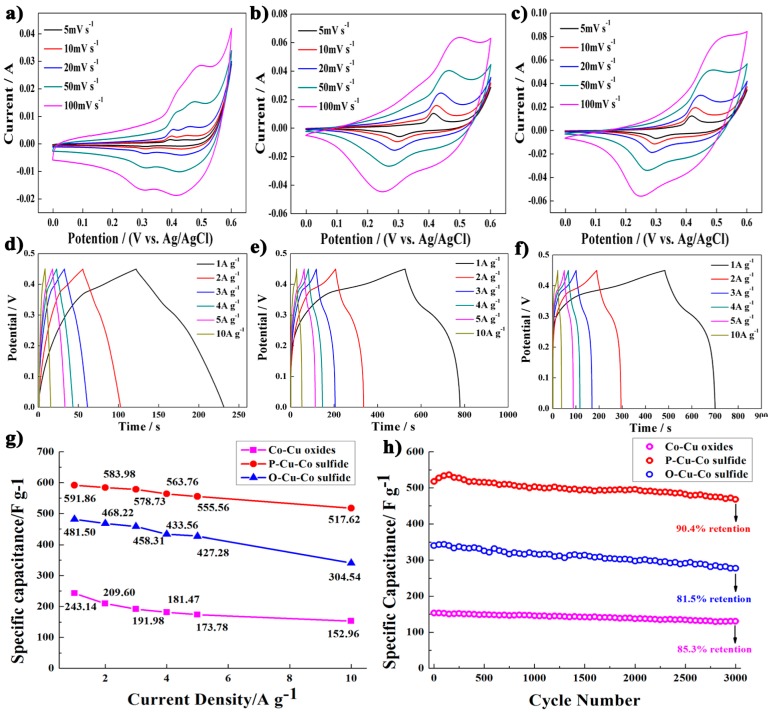
(**a**) CV curves of Cu–Co oxide; (**b**) CV curves of P–Cu–Co sulfide; (**c**) CV curves of O–Cu–Co sulfide; (**d**) GCD plots of Cu–Co oxide; (**e**) GCD plots of P–Cu–Co sulfide; (**f**) GCD plots of O–Cu–Co sulfide; (**g**) Specific capacity of Cu–Co oxide, P–Cu–Co sulfide, and O–Cu–Co sulfide; (**h**) Cycling performance of Cu–Co oxide, P–Cu–Co sulfide, and O–Cu–Co sulfide at a current density of 10 A g^−1^.

In the cyclic voltammetry (CV) curves of Cu–Co oxide (Figure 5a), it is clear that two pairs of redox peaks can be observed which indicates the typical Faradaic redox reactions occurring in the electrochemical process. At a scan rate of 100 mV s^−1^, peaks of redox pairs are respectively located at 0.30 V, 0.41 V and 0.42 V, 0.49 V which represent the conversion of Cu(I)/Cu(II) and Co–O/Co–O–OH respectively. The redox reactions of Cu–Co oxide are described by the Equations (1)–(6) [46,47,48]. The pair of redox peaks for two kinds of Cu–Co sulfides are also testified that they belong to typical electrode material for Faradaic pseudo capacitor, respectively (Figure 5b,c). Meanwhile, the oxidation peaks move to high voltage accompanied with sweep rates growing, and the reduction peaks shift into low voltage synchronously, which is conformed to quasi-reversible characteristic of redox reaction [45]. In term of the shape of CV curves, it is relatively stable when the sweep rates increase, indicating the electrode material have excellent rate capability.

Cu_2_O + 2OH^−^ ↔ 2CuO + H_2_O + 2e^−^ (1)

Cu_2_O + H_2_O + 2OH^−^ ↔ 2Cu(OH)_2_ + 2e^−^ (2)

CuOH + OH^−^ ↔ CuO+H_2_O + e^−^ (3)

CuOH + OH^−^ ↔Cu(OH)_2_ + e^−^ (4)

CoO + OH^−^ ↔ CoOOH + e^−^ (5)

CoOOH + OH^−^ ↔ CoO_2_ + H_2_O + e^−^ (6)

In Figure 5d, the plateau regions in galvanostatical charged and discharged (GCD) curves of Cu–Co oxide are not distinct because their two couples of redox peaks display slightly difference in potential. As a result, more various redox reactions for Cu–Co oxide will occur. While the GCD curves of Cu–Co sulfides exhibit distinct plateau regions which correspond to the redox peaks in the CV curves, giving the evidence of the faradaic behaviors during redox reaction process between electrode/electrolyte interfaces. Meanwhile, for comparison, the discharged times of Cu–Co sulfides are almost 2–2.5 fold to those of Cu–Co oxides at a current density of 1 A g^−1^. The specific capacitance (F g^−1^) of Cu–Co oxide and sulfides are counted using different discharge current densities as shown in Figure 5g. At current densities of 1 A g^−1^, the specific capacitance of Cu–Co oxide, P–Cu–Co sulfide and O–Cu–Co sulfide is 243, 592, and 482 F g^−1^, respectively. Surprisingly, specific capacitance of P–Cu–Co sulfide is 1.44 times higher than that of Cu–Co oxide. To increase current densities to 10 A g^−1^, specific capacitance of Cu–Co oxide, P–Cu–Co sulfide, and O–Cu–Co sulfide can maintain 153, 518, and 341 F g^−1^, separately. Fascinatedly, specific capacitance of P–Cu–Co sulfide is 2.39 times higher than that of Cu–Co oxide. In addition, when the discharge current densities increase, Cu–Co oxide only keep 63.0% retention of the capacitance, but P–Cu–Co sulfide unexpectedly hold 87.5% retention which implies good rate capability. By comparison, the specific capacitance and rate capability of Cu–Co sulfides can be superior to those of Cu–Co oxide, and P–Cu–Co sulfide have advantages over O–Cu–Co sulfide in those aspects. When the discharge current density increases, the migration and diffusion of electrolyte ions does not catch up with the reaction rate, as a result the specific values decrease gradually. This phenomenon gives the indirect evidence to the existence of the faradaic behaviors in the redox reaction [49].

**Figure 6:**
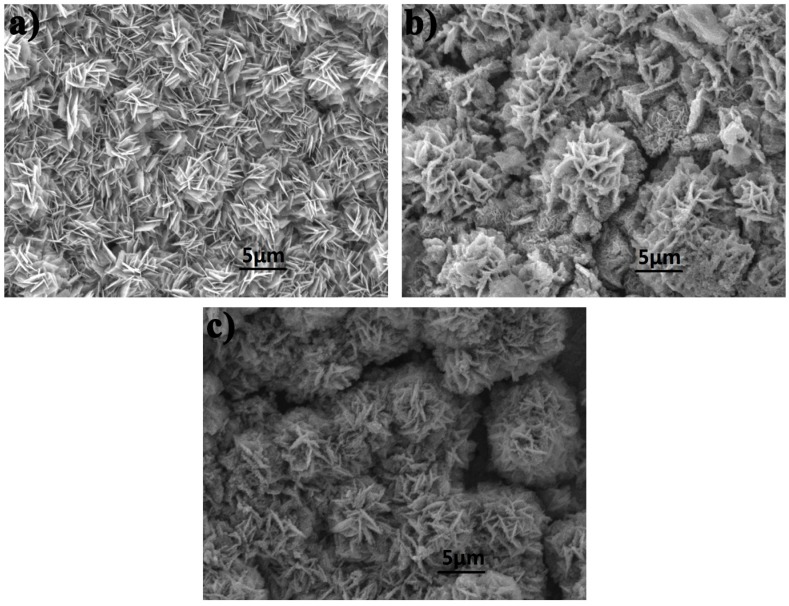
SEM images of samples after 3000 cycles at a current density of 10 A g^−1^, (**a**) Cu–Co oxide; (**b**) P–Cu–Co sulfide; (**c**) O–Cu–Co sulfide.

Moreover, repeating GCD measurements for 3000 cycles were investigated in order to test the cycle stabilities of Cu–Co oxides or sulfides at a current density of 10 A g^−1^, as shown in Figure 5h. Due to the deformation of the nanosheets structure during the repetitive charge/discharge process, the specific capacities have a gradual decline along with the cycle number increasing. After 3000 cycles, P–Cu–Co sulfide still keeps a high specific capacitance of 468 F g^−1^ with 9.6% degradation (90.4% retention) but the degradation of Cu–Co oxide is 14.7% and that of O–Cu–Co sulfide is 18.5%, which indicates that P–Cu–Co sulfide has excellent cycle stability. Figure 6 shows the morphology of electrode materials after 3000 cycles at a current density of 10 A g^−1^, the frameworks of Cu–Co oxide and sulfides still remain well after cycling, which indicate that they have good stiffness and structural stability.

## 3. Discussion

The electrochemical property of Cu–Co sulfides are meaningfully superior to that of Cu–Co oxides. However, more importantly, P–Cu–Co sulfide is surprisingly superior to O–Cu–Co sulfide in terms of electronic properties. The first factor of excellent performances of Cu–Co sulfides is that the flower-like nanosheet structure effectively provide the numerous electroactive sites bring the wide electrode-electrolyte surface/interface and facilitate the charge-transfer reactions [31,32,34]. Secondly, it is helpful to promote the conductivity property because the electroactive material connects directly to Ni foam substrate without binders [25,43]. Thirdly, Cu–Co sulfides have richer redox reaction due to the lower electronegativity of sulfur and more flexible structure owing to the substitution of oxygen to sulfur [42,43,44]. Finally, without calcination process, P–Cu–Co sulfide with low crystallinity is beneficial for faradaic reaction [26,33,36].

## 4. Materials and Methods

### 4.1. Reagents and Method

All the reagents are of analytical grade. Co(NO_3_)_2_·6H_2_O, Cu(NO_3_)_2_·3H_2_O, Na_2_S and urea were supplied by Sinopharm Chemical Reagent Co. Ltd (Beijing, China).

The as-pressed nickel foam substrates with 2 × 2 cm^2^ in size were cleaned in HCl solution and deionized water for each 15 min under ultrasonication. Typically, 1 mmol of Cu(NO_3_)_2_·3H_2_O, 2 mmol of Co(NO_3_)_2_·6H_2_O and 9 mmol of urea were dissolved in 35 mL of deionized water under magnetic stirring for 30 min. The solution was poured in 50 mL Teflon-lined stainless steel autoclave with a pretreated and weighted Ni foam, and then maintained at 120 °C for 4 h. Subsequently, the as-synthesized Cu–Co precursor loaded on Ni foams were ultrasonically cleaned with water for 1 min. The Cu–Co precursor was annealed at 350 °C in air for 2 h in order to fabricate Cu–Co oxide. The Cu–Co sulfides were prepared by the sulfuration of Cu–Co precursor or Cu–Co oxide with 10 mL 0.1 M sodium sulfide at 120 °C for 6 h. The Cu–Co sulfide obtained from precursor was recorded as P–Cu–Co sulfide, while the sulfide from Cu–Co oxide was marked as O–Cu–Co sulfide.

### 4.2. Electrochemical Measurements

In traditional three-electrode cell configuration, electrolyte is 1 M KOH aqueous solution, and the as-synthesized Cu–Co oxides or sulfides electrodes were used as the working electrodes, while Pt wire and Ag/AgCl electrodes were used as reference and counter electrodes, respectively. CV measurements were performed in the voltage range of 0 to 0.6 V (vs. Ag/AgCl). The as-synthesized Cu–Co oxides or sulfides electrodes were galvanostatically charged and discharged at different current densities between 1 and 10 A g^−1^.

## 5. Conclusions

To sum up, this work successfully synthesized uniform flower-like Cu–Co sulfide supported on nickel foam by facile two-step hydrothermal method. The differences of Cu–Co oxide, P–Cu–Co sulfide, and O–Cu–Co sulfide in morphology and electrochemical properties are systematically discussed. According to the results, P–Cu–Co sulfide electrode displays dramatically excellent electrochemical properties which performs superior specific capacity of 592 F g^−1^ at 1 A g^−1^ and 518 F g^−1^ at 10 A g^−1^, about 1.44 times and 2.39 times higher than those of Cu–Co oxide electrode, respectively. Therefore, the P–Cu–Co sulfide is expected to be a potential candidate for manipulating, controlling, and investigating novel rechargeable charge storage devices. Further works are ongoing with the research on electrochemical performance of shape-controlled hierarchical multi-metal sulfides.
